# Lymphocyte percentage and platelet count correlate with the treatment outcome to tyrosine kinase inhibitors in epidermal growth factor receptor–mutated lung adenocarcinoma

**DOI:** 10.1097/MD.0000000000021275

**Published:** 2020-07-17

**Authors:** Chi-Cheng Li, Chih-Bin Lin, Sung-Chao Chu, Wei-Han Huang, Jen-Jyh Lee, Gee-Gwo Yang, Tso-Fu Wang, Yi-Feng Wu

**Affiliations:** a Department of Hematology and Oncology; b Center of Stem Cell & Precision Medicine; c Division of Chest Medicine, Department of Internal Medicine, Hualien Tzu Chi Hospital, Buddhist Tzu Chi Medical Foundation; d Department of Medicine, College of Medicine, Tzu Chi University; e Department of Clinical Pathology, Hualien Tzu Chi Hospital, Buddhist Tzu Chi Medical Foundation, Hualien, Taiwan, ROC.

**Keywords:** lung adenocarcinoma, lymphocyte, platelet, prognosis, tyrosine kinase inhibitor

## Abstract

This observational study evaluated the treatment outcomes of clinical factors on the patients with lung adenocarcinoma with epidermal growth factor receptor mutations who received tyrosine kinase inhibitors as first-line treatment.

Patients with stage IIIb or IV lung adenocarcinoma with mutated epidermal growth factor receptor were enrolled retrospectively between March 2010 and December 2017. The hematologic markers on progression-free survival (PFS) and overall survival (OS) were analyzed.

Totally 190 patients were enrolled. In univariate analysis by hematologic markers, lower lymphocyte percentage and higher platelet count were associated with significantly poor PFS and OS. Multivariate analysis showed lower lymphocyte percentage was independent poor prognostic factors for PFS and OS. Higher platelet count was an independent poor prognostic factor for OS only.

Patients with lung adenocarcinoma receiving tyrosine kinase inhibitors with lower lymphocyte percentage and higher platelet count had poorer prognoses compared with other patients.

## 1 Introduction

According to the World Health Organization, lung adenocarcinoma is the major causes of mortality.^[1]^ Epidermal growth factor receptor (EGFR) tyrosine kinase inhibitors (TKIs) as the first-line therapy were used for treatment of EGFR-mutant lung adenocarcinoma for more than 10 years. And the response rates, progression-free survival (PFS) and overall survival (OS) had been improved by TKIs significantly.^[2]^ Although the response rate is high with EGFR TKIs as first-line treatment, there are still some patients with poor prognosis. Several biomarkers, including CA125 (cancer antigen 125), CEACAM (carcinoembryonic antigen-related cell adhesion molecule), neuron-specific enolase, and CYFRA21-1 (cytokeratin-19 fragments), all showed limited sensitivity and specificity.^[3]^

Evidence by previous studies had been showed changes in a white blood count and platelet in cancer patients associated with the disease severity and survival.^[4–6]^ Lymphocytes also played critical roles in promoting systemic immune responses against tumors, and lymphocytopenia is associated with poor outcomes in many malignancies.^[7,8]^ High expression of CD8+ T lymphocytes, which predicts a favorable prognosis in lung adenocarcinoma was reported.^[9]^ Platelet played another important role in cancer prognosis, too. Thrombocytosis has been found which is associated with poorer cancer prognosis. Shorter OS rates observed for patients with many malignancies, included ovarian cancer,^[5]^ lung cancer,^[4]^ and breast cancer^[6]^ which was related to thrombocytosis at the time of diagnosis, and poor prognoses of patients with colorectal cancer^[10]^ and renal cancer^[11]^ before surgical therapy are related to high platelet counts. Sylman et al reported that platelet count is also a predictor of metastasis in patients with cancer.^[12]^ On the other hand, systemic inflammation also plays a role in cancer prognosis.^[13]^ Inflammatory mediators are involved in cancer progression with apoptosis, angiogenesis, and DNA damage.^[14]^ The markers included the neutrophil-to-lymphocyte ratio (NLR) and platelet-to-lymphocyte ratio (PLR). Higher NLR or PLR has been reported to predict shorter PFS and OS in many solid cancers.^[15–17]^

Awareness of newer prognostic factors might provide a potential direction for further improvement in treatment for EGFR-mutated non–small-cell lung cancer, especially adenocarcinoma, but no study has focused on hematologic and inflammatory markers in EGFR-mutant lung adenocarcinoma. In this study, we evaluated the effects of hematologic and inflammatory factors on the treatment outcomes of patients with advanced or metastatic lung adenocarcinoma with active EGFR mutations. All patients received TKIs as the first-line treatment.

## 2 Patients and methods

### 2.1 Patients and data collection

**Figure 1:**
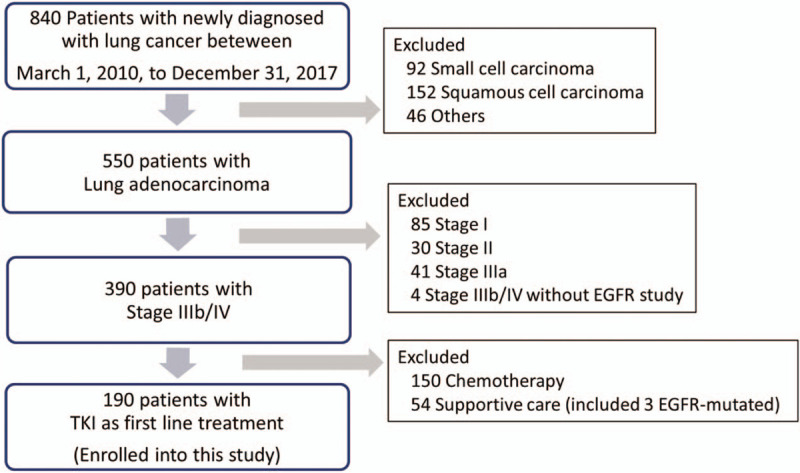
Study flowchart.

From March 1, 2010, to December 31, 2017, totally 840 patients were diagnosed newly with lung cancer in Buddhist Tzu Chi General Hospital, Hualien, Taiwan. There were 550 patients with adenocarcinoma, and 394 patients with stage IIIb or IV. Three hundred ninety patients with stage IIIb or IV lung adenocarcinoma had EGFR study (4 patients did not have EGFR study). In 390 patients, there were 193 patients showed EGFR-mutated, and 3 patients with EGFR–mutated received supportive care only. We enrolled the patients with stage IIIb or IV adenocarcinoma and received tyrosine kinase inhibitors as first line treatment only in this retrospective study. (Fig. 1)

According to the World Health Organization pathology classification, lung adenocarcinoma was confirmed by biopsy. All patients received serial imaging studies at the initial diagnosis for staging, including computed tomography, whole-body bone scan, positron emission tomography scan, and brain imaging. Tumor staging was recorded by the seventh American Joint Committee on Cancer staging system. All the patients had an EGFR mutation examination of the tumor specimen, and the results showed active EGFR mutations in exons 18, 19, 20, or 21 in all patients. And then they received EGFR TKIs as first-line therapy, including afatinib, erlotinib, or gefitinib. The choice of EGFR TKI was by the decision of their attending physicians. Baseline clinical characteristics were determined through a retrospective chart review, including age at diagnosis, sex, staging, smoking status, mutation type, and TKIs used. Malignant pleural effusion was diagnosed by either pleural effusion cytology or a pleural biopsy. Complete blood counts, including total leukocyte counts with a different count, hemoglobin, and platelet count, were also recorded. NLR and PLR were also calculated from the data of complete blood counts.

Mutational analysis of EGFR gene was done as described in a previous study.^[18]^ Briefly, formalin-fixed, paraffin-embedded tissues were used. An EGFR RGQ Kit (Qiagen, Hilden, Germany) was used for the analysis of mutations in EGFR, which utilizes amplification refractory mutation-specific polymerase chain reaction and Scorpion technologies for detection and direct sequencing.

PFS was recorded as the duration between the start of TKI treatment and the date of progression. And OS was defined as the duration from the start of TKI treatment to the time of all-cause death. All enrolled patients were followed up until death or the end of December 2018. This retrospective study was approved by the Institutional Review Board of Buddhist Tzu Chi General Hospital (IRB108–48-B). Informed written consent was waived because the study was a retrospective data analysis.

### 2.2 Statistical analyses

MedCalc (Mariakerke, Belgium) was used for analysis with median and hazard ratios (HRs) and 95% confidence intervals (CIs). Kaplan–Meier curves and the log-rank test were used for analysis of PFS and OS. Univariate and multivariate analyses were executed using Cox proportional-hazards regression, and all variables were included for multivariate analysis to assess the effect of each variable after univariate analyses. Stepwise variable selection was used to develop a reduced multivariate model, including variables with *P* < .1 and removing variables with *P* > .2. Because of above, many factors, for example age, gender, stage (IIIb vs IV), WBC count, Hb, did not showed the *P*-value in multivariate analysis due to no significant difference in univariate test. Two-sided with the level of statistical significance set at *P* < .05 was used in all results.

## 3 Results

### 3.1 Clinical features of patients

Overall, 190 patients, including 87 male (45.8%) and 103 female (54.2%), were enrolled. The median follow-up duration was 17.9 months (range, 0.5–91.3 months). The clinical features of this study population, such as age, sex, smoking history, stage, EGFR mutation, drugs used, malignant pleural effusion, and brain metastasis, are summarized in Table 1. The median age was 70 years (range, 42–95). In all patients, adenocarcinoma was confirmed by pathologists. Twenty-one (11.1%) and 169 (88.9%) patients were at TNM stages IIIb and IV, respectively. As the type of EGFR mutation, 89 patients (46.8%) were confirmed with mutation of L858R, 89 (46.8%) with exon 19 deletions, and the remaining 12 (6.3%) had other mutations that were sensitive to TKI treatment by previous reports. The 12 uncommon active mutations of EGFR were 7 for L871Q in exon 21, 1 for L858 M, 1 for S768I in exon 20, 1 for G719X in exon 18, and 2 for 2-point mutations with E709G/L858R and G719X/L861Q.

**Table 1:**
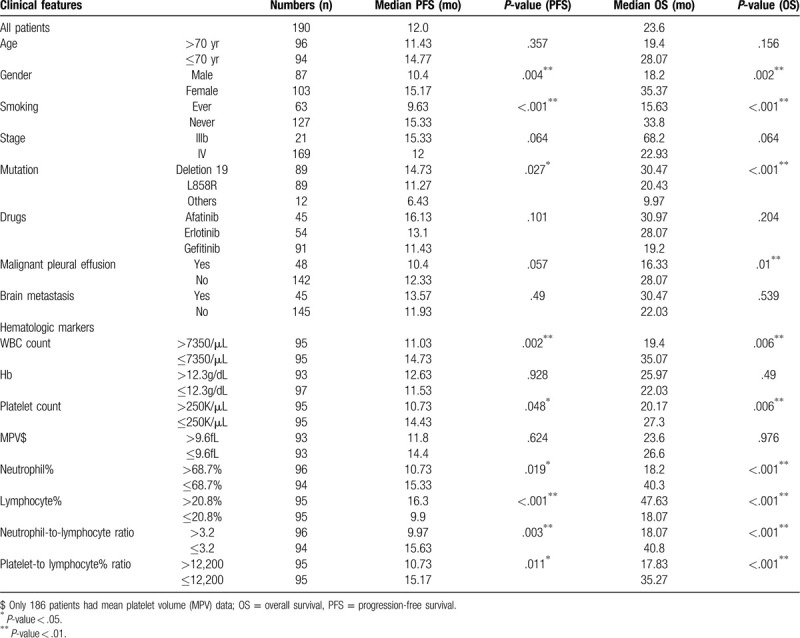
Median progression-free survival and overall survival of patients with specific clinical features.

The TKIs used for all patients, including gefitinib in 91 (47.9%) patients, erlotinib in 54 (28.4%), and afatinib in 45 (23.7%). 66.8% patients reported being never smokers. Malignant pleural effusion at initial diagnosis was observed in 48 (25.3%) patients. Among the patients, 152 (80%) had disease progression, and 110 (57.9%) died during follow-up.

### 3.2 Clinical features versus lung adenocarcinoma under TKI treatment

Univariate analysis results showed that the male (median PFS: 10.4 vs 15.17 months, *P* = .0036; median OS: 18.2 vs 35.37 months, *P* = .002) and ever smoking (median PFS: 9.63 vs 15.33 months, *P* < .001; median OS 15.63 vs 33.8 months, *P* < .001) were associated with poor PFS and OS. About EGFR mutation types, exon 19 deletion had better PFS and OS than L858R or others did (median PFS: 14.73, 11.27, vs 6.43 months, *P* = .027; median OS: 30.47, 20.43, vs 9.97 months, *P* < .001). Malignant pleural effusion at the time of initial diagnosis was associated with poor OS only, not PFS. (median PFS: 10.4 vs 12.33 months, *P* = .057; median OS: 16.33 vs 28.07 months, *P* = .01).

Under multivariate analysis with a stepwise model, the results revealed that ever smoking was independent poor prognostic factors for PFS and OS (PFS: HR = 0.56, 95% CI: 0.40–0.79, *P* = .001; OS: HR = 0.55, 95% CI: 0.37–0.83, *P* = .004). EGFR exon 19 deletion was independent better prognostic factors for PFS and OS (PFS: HR = 1.35, 95% CI: 1.03–1.76, *P* = .029; OS: HR = 1.65, 95% CI: 1.20–2.29, *P* = .002). Malignant pleural effusion at the time of initial diagnosis was associated with poor OS still (OS: HR = 0.63, 95% CI: 0.42–0.95, *P* = .029). The detailed data are presented in Tables 1 and 3.

**Table 2:**
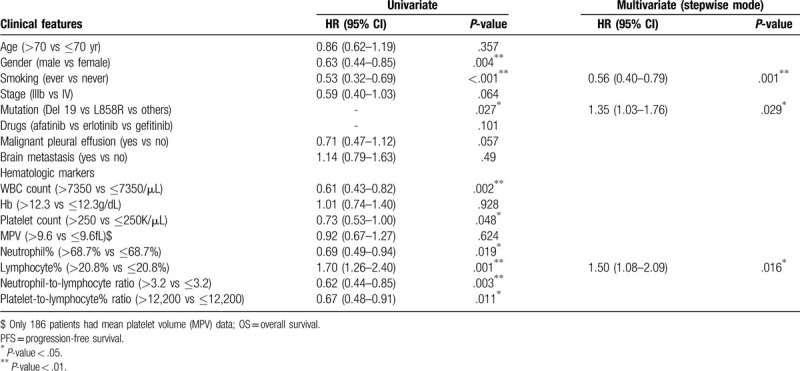
Analyses for the relationship between clinical features and progression-free survival.

**Table 3:**
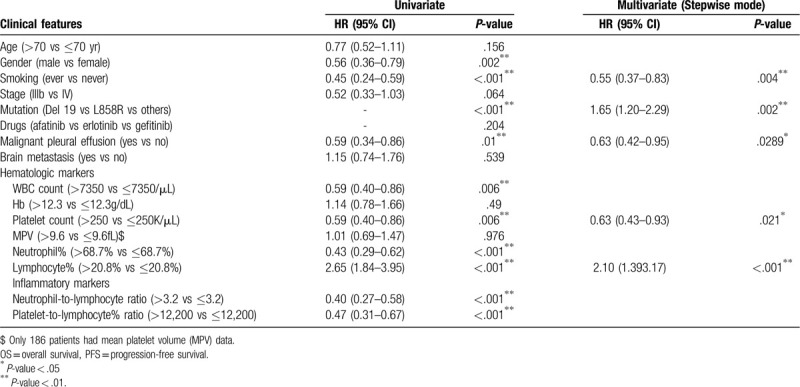
Analyses for the relationship between clinical features and overall survival.

### 3.3 Hematologic markers versus lung adenocarcinoma under TKI treatments

**Figure 2:**
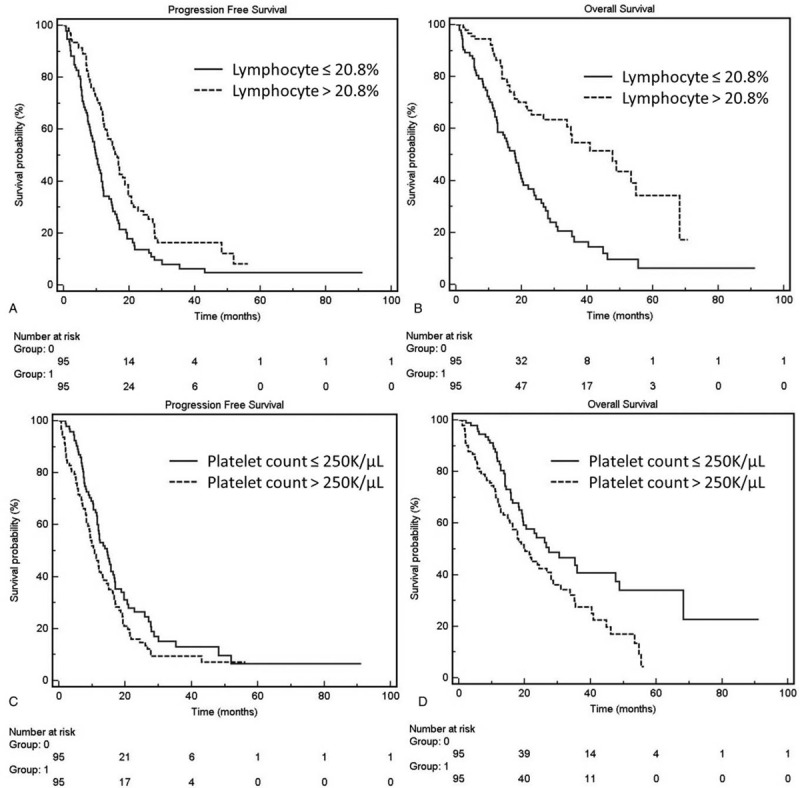
Kaplan–Meier curves of progression-free survival (PFS) and overall survival (OS). Kaplan–Meier curves of PFS and (A) and OS (B) constructed based on the lymphocyte percentage (PFS, 9.9 vs 16.3 months, *P* = .001; OS, 18.07 vs 47.63 months, *P* < .001). Kaplan–Meier curves of PFS (C) and OS (D) constructed based on the platelet count (PFS, 14.43 vs 10.73 months, *P* = .048; OS, 27.3 vs 20.17 months, *P* = .006).

Lower leukocyte count (lower than 7350/μL vs higher, median PFS: 14.73 vs 11.73 months, *P* = .002; median OS: 35.07 vs 19.4 months, *P* = .006) and lower platelet count (lower than 250,000/μL vs higher, median PFS: 14.43 vs 10.73 months, *P* = .048; median OS: 27.3 vs 20.17 months, *P* = .006) were significantly associated with a better prognosis in the univariate analysis. Higher lymphocyte percentage (higher than 20.8% vs lower, median PFS: 16.3 vs 9.9 months, *P* < .001; median OS: 47.63 vs 18.07 months, *P* < .001) was also significantly associated with better PFS and OS (Fig. 2). In the univariate analysis, the cutoff levels of NLR and PLR were established based on median values: 3.2 and 12,200. Lower NLR (median PFS: 15.63 vs 9.97 months, *P* = .003; median OS: 40.8 vs 18.07 months, *P* < .001) and lower PLR (median PFS: 15.17 vs 10.73 months, *P* = .011; median OS: 35.27 vs 17.83 months, *P* < .001) were associated with better PFS and OS.

In the multivariate analysis with a stepwise model, only higher lymphocyte percentage was significantly associated with better prognosis in PFS (HR = 1.50, 95% CI: 1.08–2.09, *P* = .016). Regarding OS, higher lymphocyte percentage (HR = 2.10, 95% CI: 1.39–3.17, *P* < .001) was also associated with a better prognosis, but higher platelet count (HR = 0.63, 95% CI: 0.43–0.93, *P* = .021) was associated with a significantly poorer prognosis, too.

## 4 Discussion

Lung cancer is a prominent global health burden that causes approximately 1.5 million annual deaths by previous reports.^[19]^ The prognosis of patients with advanced-stage lung adenocarcinoma with genotype-driven mutations has improved owing to target therapy administration,^[20,21]^ including in-frame deletions in exon 19 (Del19) or exon 21 substitution of leucine for arginine (L858R).^[22,23]^ Patients with lung adenocarcinoma who harbor these classical mutations have high objective response rates to the first-generation reversible adenosine triphosphate-competitive EGFR TKIs, including gefitinib and erlotinib, and second-generation irreversible TKI, including afatinib. Despite the high response rates (52.7%–83%) of TKIs used in treating patients with stage IIIb or IV lung cancer with active EGFR mutations, such patients eventually succumb to this disease; To further improve the outcome, more aggressive treatment might be necessary for some patients. Therefore, methods for predicting prognosis have become increasingly critical. In this study, we demonstrated that lower lymphocyte percentage predicted poor PFS and OS and higher platelet count predicted poor OS in patients with active EGFR mutations receiving TKIs as the first-line treatment.

Several retrospective studies have shown longer PFS after TKI treatment in patients harboring EGFR with exon 19 deletions than in those with exon 21 mutations. Won et al reported that 87 patients with exon 19 deletions had longer PFS following EGFR TKI treatment.^[24]^ Zhou et al also reported that 219 patients with exon 19 deletions had longer survival times significantly.^[25]^ In our data, the patients with exon 19 deletions had better PFS and OS than did those with L858R. Previous studies have shown that malignant pleural effusion at initial diagnosis^[26]^ is associated with poor OS in lung cancer patients. A recent study proposed that cancer stem cells in pleural effusion contribute to the metastatic cascade through the epithelial-mesenchymal transition, anoikis, and adaptation in the microenvironment. This result may explain the high therapeutic failure rates if the patients have malignant pleural effusion.^[27]^ Our results also showed the patients with malignant pleural effusion would have a poor prognosis. In addition to malignant pleural effusion and EGFR mutation sites, sex and smoking history are common prognostic factors in cancers.^[18]^ Our results show that male and a smoker were positively associated with poor prognosis by univariate analysis but not by multivariate analysis.

In our study, an increase in lymphocyte percentage along with a related increase in leukocyte count was associated with a better prognosis. Because lymphocytes are critical in promoting systemic immune responses against tumors, lymphocytopenia is associated with poor outcomes in many solid cancers.^[7,8]^ Cytotoxic T lymphocytes elicit adaptive cellular immunity against tumor cells,^[28]^ and Ye et al found that high expression of CD8+ T lymphocytes predicts better prognosis in patients with lung adenocarcinoma.^[9]^ In our study, patients with a lymphocyte percentage of >20.8% had a better prognosis than did other patients. Higher lymphocyte percentage and lower lymphocyte percentage were associated with median OS periods of 40.63 and 18.07 months, respectively, and these were significantly different between univariate and multivariate analyses.

High platelet count has been reported with a poor prognosis in various cancers.^[29]^ The mechanism may be related to thymidine phosphorylase, which is a platelet-derived endothelial cell growth factor with potent angiogenic activity. An increase in thymidine phosphorylase levels may be associated with a poor prognosis in various solid tumor tissues.^[30]^ Thrombocytosis with a negative prognostic value in lung cancer has been reported before.^[31]^ However, no study has found that higher platelet count is associated with a poorer prognosis in only lung adenocarcinoma with EGFR mutations under TKI treatment. In our results, 190 patients with EGFR-mutated lung adenocarcinoma under TKI treatment demonstrated that patients with higher pretreatment platelet counts had shorter PFS and OS. Notably, under multivariate analysis, higher platelet count was found to affect only OS and not PFS. TKI treatment may be able to overcome the effects of platelet count; additional studies are required to elucidate this hypothesis.

Some studies showed lower NLR, or PLR ratio exhibited better PFS and OS in non-small-cell lung cancer patients.^[32,33]^ Inflammation may contribute to tumor initiation through genetic mutations, genomic instability, and epigenetic modifications, and stimulate angiogenesis, causes immunosuppression, and promotes the formation of microenvironments, and metastatic spread.^[34]^ Accordingly, the close association between increased systemic inflammatory responses, including NLR and PLR, and poor prognosis identified in our study may be associated with inflammatory in cancer cells.

The limitation of this study is the retrospective analysis of an observational database, and all enrolled patients were obtained from a single institution.

In conclusion, in our study of patients with stage IIIb or IV lung adenocarcinoma with EGFR mutations who received TKIs as the first-line treatment, a lower lymphocyte percentage, and higher platelet counts were significantly associated with shorter PFS and OS. Stepwise multivariate Cox regression analysis also showed that lower lymphocyte percentage was an independent poor prognostic factor for both PFS and OS, but a higher platelet count was only for OS. Further research is necessary to confirm the possible mechanism of poor prognoses.

## Author contributions

**Conceptualization:** Chi-Cheng Li, Yi-Feng Wu.

**Data curation:** Chih-Bin Lin, Sung-Chao Chu, Jen-Jyh Lee, Gee-Gwo Yang, Tso-Fu Wang.

**Methodology:** Wei-Han Huang.

**Formal analysis:** Tso-Fu Wang, Yi-Feng Wu.

**Supervision:** Yi-Feng Wu.

**Writing – original draft:** Chi-Cheng Li, Yi-Feng Wu.
